# Evolution of a Genome-Encoded Bias in Amino Acid Biosynthetic Pathways Is a Potential Indicator of Amino Acid Dynamics in the Environment

**DOI:** 10.1093/molbev/msu225

**Published:** 2014-08-12

**Authors:** Rick A. Fasani, Michael A. Savageau

**Affiliations:** ^1^Department of Biomedical Engineering and Microbiology Graduate Group, University of California, Davis

**Keywords:** cognate bias, genomes, proteomes, environmental stress, stringent response, cell fate

## Abstract

Overcoming the stress of starvation is one of an organism’s most challenging phenotypic responses. Those organisms that frequently survive the challenge, by virtue of their fitness, will have evolved genomes that are shaped by their specific environments. Understanding this genotype–environment–phenotype relationship at a deep level will require quantitative predictive models of the complex molecular systems that link these aspects of an organism’s existence. Here, we treat one of the most fundamental molecular systems, protein synthesis, and the amino acid biosynthetic pathways involved in the stringent response to starvation. These systems face an inherent logical dilemma: Building an amino acid biosynthetic pathway to synthesize its product—the cognate amino acid of the pathway—may require that very amino acid when it is no longer available. To study this potential “catch-22,” we have created a generic model of amino acid biosynthesis in response to sudden starvation. Our mathematical analysis and computational results indicate that there are two distinctly different outcomes: Partial recovery to a new steady state, or full system failure. Moreover, the cell’s fate is dictated by the cognate bias, the number of cognate amino acids in the corresponding biosynthetic pathway relative to the average number of that amino acid in the proteome. We test these implications by analyzing the proteomes of over 1,800 sequenced microbes, which reveals statistically significant evidence of low cognate bias, a genetic trait that would avoid the biosynthetic quandary. Furthermore, these results suggest that the pattern of cognate bias, which is readily derived by genome sequencing, may provide evolutionary clues to an organism’s natural environment.

## Introduction

Predicting or measuring the natural microenvironment of an organism is a complex and challenging task (Savageau 1983; Ward et al. 1998; Xu 2006). In contrast, sequencing an organism’s genome has become routine, and the scientific community continues to sequence organisms from varied environments at an increasing pace, yet the vast majority of those organisms cannot be cultured by current methods, in part because their natural environment is unknown (Curtis and Sloan 2005; Xu 2006). Genomic data are clearly outpacing environmental data, but the sequences themselves may provide information about the environment from which they were taken. Recent statistical analyses of overall amino acid composition across organisms indicate that the environment is a major evolutionary influence (Chen et al. 2013; Moura et al. 2013). More specifically, the cognate bias hypothesis (Alves and Savageau 2005) suggests that nutritional stress places evolutionary pressure on the composition of the enzymes in the amino acid biosynthetic pathways. (The “cognate amino acid” refers to the amino acid produced by the corresponding biosynthetic pathway, and the “cognate bias” refers to the number of cognate amino acids in the enzymes of the corresponding biosynthetic pathway relative to their number in the proteome of the organism.)

The bacterial response to nutritional stress, the well-known stringent response, has been studied for over five decades. In 1961, it was shown that amino acid starvation inhibited the accumulation of stable RNA, and the locus responsible was christened RC, or the RNA Control gene (Stent and Brenner 1961), later renamed *relA* in reference to the relaxed response of the mutant phenotype (Friesen et al. 1974). Today, it is clear that the stringent response is a general reaction to stress and starvation that is conserved across species (Draper 1996; Harris et al. 1998; van der Biezen et al. 2000; Chatterji and Ojha 2001), and is characterized by increased levels of guanosine tetraphosphate (ppGpp) (Cashel 1969; Wendrich et al. 2002), which has at least 75 known effects in *Escherichia coli*, including decreased rRNA and tRNA transcription, decreased growth rate, and increased expression of the biosynthetic enzymes for many amino acids (Draper 1996; Magnusson et al. 2005; Potrykus and Cashel 2008; Dalebroux and Swanson 2012). However, the stringent response may not be enough to protect the cell from the shock of starvation. Part of the response is the upregulation of amino acid biosynthetic pathways, but the situation creates a potential catch-22. The missing amino acid could hold up the construction of the enzymes needed to create more of their cognate amino acid, a stalemate from which the cell might not recover. A logical evolutionary defense would be to remove the vulnerability—to bias the biosynthetic enzymes against the use of their cognate amino acid.

Our first hint that organisms might evolve such a molecular mechanism came in the early days of protein sequencing when tryptophane synthetase, an enzyme in the tryptophan biosynthetic pathway, was sequenced and the alpha subunit was found to contain no tryptophan (Yanofsky 1988). Hardly conclusive, it took many years and a new technology to begin testing the hypothesis more generally. Additional support for what we termed the cognate bias hypothesis was obtained from genomic data for three well-studied bacteria: Two enteric bacteria, *E. coli* and *Salmonella enterica* (serovar Typhimurium), and the soil-dwelling bacteria *Bacillus subtilis* (Alves and Savageau 2005). The results suggested a bias toward fewer cognate amino acids in certain amino acid biosynthetic pathways and a profile of bias across amino acids that differed between the two groups, suggesting a possible correlation with the organisms’ ecological niches. The cognate bias hypothesis was recently tested and confirmed using a few organisms from the other domains of life: *Methanococcus jannaschii*, *Saccharomyces cerevisiae*, and *Homo sapiens* (Perlstein et al. 2007; Meiler et al. 2012). However, the number of organisms examined to date is too few to provide for a meaningful statistical test.

Complete genome sequences are now available for more than 1,800 microorganisms. Thus it is an opportune time to go beyond correlations and comprehensively examine the cognate bias hypothesis, with an analysis of the underlying molecular mechanism using a kinetic model of protein synthesis and amino acid starvation, in order to provide a stronger molecular link between the genomic evidence of amino acid composition and the environmental dynamics of amino acid availability.

In particular, we are interested in the environment’s effect on the proteome, in terms of three classes of amino acids: 1) For amino acids the organism is never required to create, it could dispense with the biosynthetic pathway entirely (e.g., as occurs with obligate intracellular parasites that receive the amino acid from their host, and with humans that receive their essential amino acids from their diet); 2) for amino acids the organism is always required to create, it could dispense with the regulation and synthesize the amino acids constitutively, without regard for cognate bias; and 3) for all other environments, regulation would be advantageous, and a compensating cognate bias would likely exist for amino acids that experience the most frequent and extreme fluctuations in the organism’s natural environment. In order to study the last case more rigorously, we have created a generic model of amino acid biosynthesis and regulation in response to sudden starvation. The results indicate that there are two distinctly different outcomes—partial recovery or full failure—that are dictated by the cognate bias, the number of cognate amino acids in the corresponding biosynthetic pathway relative to the average number of that amino acid in the proteome. Furthermore, we mine the abundant genomic data that are currently available and reveal statistical evidence of cognate bias. The results describe how the natural environment of an organism—or more precisely, the stresses and strains to which the organism is exposed—may leave a genetic fingerprint.

## Results

### Model of Translation during Starvation

**Fig. 1.**
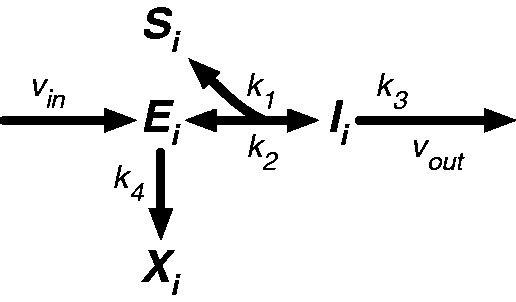
Model of a single branch point in translation. At step *i*, the advancement of the ribosome is modeled by a single enzymatic reaction. The required aa-tRNA *S_i_* enters the A-site of the ribosome–mRNA–peptide complex *E_i_*, to create a new intermediate complex *I_i_*. The process is reversed if the aa-tRNA leaves the A-site. Otherwise, the amino acid is irreversibly linked to the growing peptide chain, advancing the complex to *E_i_*_ + 1_. In the absence of aa-tRNA, the complex can dissociate from the mRNA, creating the aborted complex *X_i_*. *k*_1_, rate constant of the appropriate aa-tRNA entering the A-site; *k*_2_, rate constant of the appropriate aa-tRNA leaving the A-site; *k*_3_, rate constant of amino acid *i* being incorporated into the growing peptide chain; *k*_4_, rate constant of the ribosome aborting and dissociating from the mRNA; *v*_in_, rate of ribosomes advancing to the current position; *v*_out_, rate of ribosomes proceeding to the next position.

Before describing a larger model of amino acid biosynthesis and regulation, we present a model of translation that accounts for the effect of starvation, or more specifically for a decreased supply of the cognate amino acid of interest. Mathematical models of the translational process have been created, but they are too detailed to be tractable within our larger model of regulation (Gromadski and Rodnina 2004; Elf and Ehrenberg 2005; Gilchrist and Wagner 2006; Fluitt et al. 2007; Shah and Gilchrist 2010; Brackley et al. 2011, 2012). Here, we consider a somewhat simpler approach. Translation proceeds through three well-known steps (Alberts et al. 2002). First, the ribosome, which is attached to the mRNA and a growing peptide chain, exposes an empty A-site. Second, a charged, aminoacyl-tRNA (aa-tRNA) fills the empty A-site. In fact, the aa-tRNA may leave the A-site, returning the system to the first step. Finally, the ribosome incorporates the amino acid into the peptide chain, discharges the uncharged tRNA, and advances. The sequence of steps is reminiscent of an enzymatic reaction and has in fact been modeled as such in the past by Elf and Ehrenberg (2005). However, they consider the incorporation of an amino acid in isolation—a single instance of those three steps—and use the result to represent the average rate of amino acid consumption in a larger model of global protein synthesis. Here, we consider the sequential incorporation of every amino acid in the protein as a longer sequence of enzymatic reactions, and consequently the combined impact of starvation at multiple steps in the synthesis of a single protein. When modeling translation as an enzymatic reaction, the ribosome–mRNA complex represents the catalyst. The transition between the first and second steps is treated as a reversible reaction between a complex with an empty A-site and an intermediate complex with an aa-tRNA in the A-site. The transition between the second and third steps—the addition of the amino acid to the peptide chain and the advancement of the ribosome—is treated as the essentially irreversible step in the enzymatic reaction. Figure 1 depicts the transitions between states. We assume that the concentration of aa-tRNA is coupled to the concentration of the free amino acid pool, so that when the supply of amino acid decreases, the concentration of aa-tRNA decreases proportionally, and the rate of its incorporation into the A-site decreases. In the limiting case where none of the particular amino acid is available, the ribosome remains stalled with an empty A-site. We assume that the longer the ribosome is stalled, the more likely it is to prematurely terminate translation and dissociate from the mRNA, which is consistent with models of nonsense errors produced by frameshift, ribosomal drop-off, or release factors (Jørgensen and Kurland 1990; Gilchrist and Wagner 2006; Shah and Gilchrist 2010) and models of ribosome rescue by transfer-messenger RNA (Roche and Sauer 1999; Moore and Sauer 2007; Keiler 2008).

**Fig. 2.**
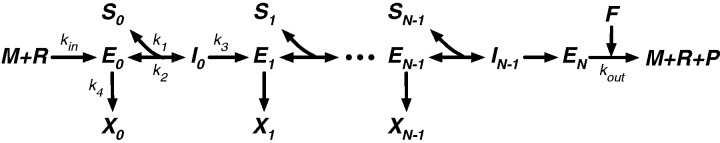
Model of translation as an enzymatic process. The translation of a full protein is modeled by a series of enzymatic reactions, each as shown in figure 1. Free ribosome *R* binds to mRNA *M* and initiates translation. At each step *i*, the required aa-tRNA *S_i_* is added to the ribosome–mRNA–peptide complex *E_i_* that already has a peptide chain of length *i*. The final complex *E_N_* is released by release factor *F* to create free ribosome *R*, mRNA *M*, and protein *P*. *k*_in_, rate of translational initiation; *k*_out_, rate of protein release. *I_i_*, *X_i_*, and *k*_1_–*k*_4_ are described in figure 1.

The complete translation of a protein with *N* amino acids can be modeled as a linked chain of *N* enzymatic reactions, as shown in figure 2. The entire process begins with initiation, when the ribosome binds to the mRNA, and ends with the release of the completed protein, which requires the presence of release factors. If all of the amino acid concentrations are high, the rate of abortion becomes vanishingly small, and the entire process can be viewed as an unbranched pathway, implying that at steady state, what goes in must come out. Assuming the concentrations of ribosome and all ancillary factors are constant or saturating, then the steady-state rate of protein production, without starvation and abortion, is simply $v_{\text{out}}=k_{\text{in}}M$, or proportional to the concentration of the specific mRNA. Nearly all models of biosynthetic gene circuits treat the rate of protein synthesis as proportional to the concentration of mRNA, and there is good evidence in bacterial literature to support this (Guet et al. 2008). On the other hand, if the supply of a particular amino acid is significantly decreased, then the ribosome will stall at each point that requires that amino acid (Pedersen 1984; Varenne et al. 1984; Sørensen et al. 1989), increasing the rate of abortion and slowing the overall rate of protein production. The exact rate of protein production can be determined by an inductive argument. In the simplest case, where the protein only requires a single amino acid that is in short supply, the process has a single branch point at step *i*, as shown in figure 1. It can be shown that at steady state, *v*_out_ at step *i* is a function of *v*_in_ and the limiting amino acid concentration, or

(1) $$v_{\text{out}}=v_{\text{in}}\left(\frac{S_{i}}{S_{i}+K_{m}}\right),$$

where

(2) $$K_{m}=\frac{k_{\text{4}}}{k_{\text{3}}}\left(\frac{k_{\text{2}}+k_{\text{3}}}{k_{\text{1}}}\right).$$

More intuitively, *K_m_* is the amino acid concentration for half-maximal velocity through step *i*. *v*_in_ is a result of the proceeding steps, which in this case form an unbranched pathway, and is therefore $k_{\text{in}}M$ at steady state. The remaining steps also form an unbranched pathway, meaning that the overall rate of protein production is equal to the velocity through step *i*.

For a protein that requires more than one of the limiting amino acid, *v*_out_ at the first occurrence is identical to the case with one. *v*_out_ at the second occurrence is equal to the output of the proceeding steps multiplied by the same attenuating factor. As such, for a protein that requires *n* of the limiting amino acid, the overall rate of protein production is

(3) $$v_{\mathrm{out}}=k_{\text{in}}M{\left(\frac{S}{S+K_{m}}\right)}^{n},$$

where *S* is the concentration of the limiting amino acid. The implication of equation (3) is dramatic. Considering the average protein in *E. coli* contains approximately 300 amino acids, and assuming the 20 amino acids are equally represented, *n* is on the order of 15, making translation ultrasensitive to the limiting amino acid concentration. If the amino acid concentration drops below some critical threshold, *K_m_*, then the rate of translation will practically halt.

The submodel above captures the essential features needed in our larger model of amino acid biosynthesis and regulation in the next section. One could conceivably add more detail, and attempt to account for such factors as tRNA abundance, specific constants for the binding of amino acid to various tRNAs and the binding of tRNA anticoding sites to codons in mRNA, salt and pH concentrations, or any other physical–chemcial aspect of the intracellular milieu that differs according to cell type (e.g., high pH in extreme halophilic archaea vs. low pH in halophobic bacteria). However, such detailed models would become so complex that analysis would be difficult if no precluded, most values of the parameters would not be available for most organisms, and even if these obstacles were overcome in a particular case, the results would not generalize to other systems.

### Model of Amino Acid Biosynthesis and Regulation

**Fig. 3.**
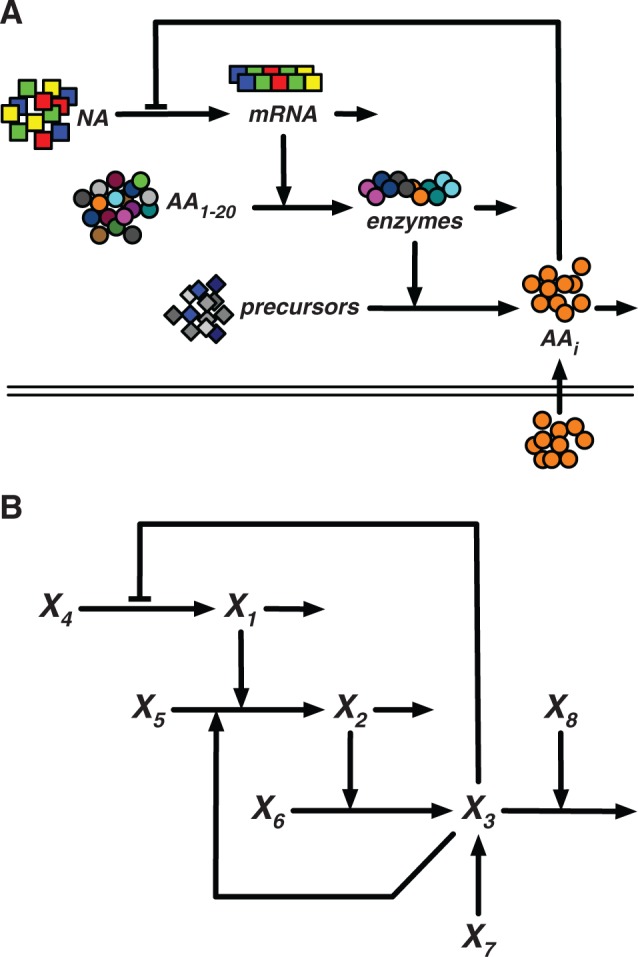
Model of amino acid biosynthesis and regulation during starvation. (*A*) Model illustration of amino acid biosynthesis and regulation with an external supply. NA, nucleic acid precursors; mRNA, messenger RNA for the enzymes of the amino acid biosynthetic pathway; AA_1–20_, free amino acid; AA*_i_*, free cognate amino acid. (*B*) Abstract model of species concentrations and interactions used here. *X*_1_, mRNA; *X*_2_, critical enzyme of the amino acid biosynthetic pathway; *X*_3_, free cognate amino acid; *X*_4_, nucleic acid precursors; *X*_5_, protein precursors; *X*_6_, cognate amino acid precursors; *X*_7_, external cognate amino acid; *X*_8_, total cellular mRNA.

Mathematical models of amino acid biosynthetic systems have been developed in the past, in many cases for specific systems, such as Trp biosynthesis in *E. coli* (Xiu et al. 2002; Alves and Savageau 2005; Elf and Ehrenberg 2005). Many of these models tend to be complex with idiosyncratic features that do not readily generalize to other systems, as discussed above for our submodel. For instance, the pathways may have different numbers of enzymes with very different kinetic properties. Rather, we require relatively simple models that nevertheless retain the essential generic character of amino acid biosynthetic systems, can be readily analyzed to make testable predictions, and can be used to elucidate general design principles. For example, models of inducible and repressible pathways, very similar to the one developed below, were used to make predictions regarding the coupling of expression in elementary gene circuits; the resulting predictions were confirmed experimentally in over 50 specific cases and the predicted coupling rules are now established as a general design principle (Hlavacek and Savageau 1996, 1997; Wall et al. 2003, 2004). Figure 3 depicts our model of amino acid biosynthesis, one that includes the transcription and translation of enzymes in the biosynthetic pathway, as well as the synthesis of the cognate amino acid. Feedback repression of the biosynthetic enzymes, which is a prominent control mechanism in bacteria (Neidhardt et al. 1996), is also included, as is the ability to import amino acid from the external environment. As was shown in the previous section, the rate of translation of the biosynthetic enzymes depends on the concentration of the free cognate amino acid, which is also depleted by cellular demand.

The model is mathematically described by a conventional system of ordinary differential equations (ODEs):

(4) $$\frac{\text{d}X_{\text{1}}}{\text{d}t}=\alpha_{\text{1}}X_{\text{4}}\left(\frac{\sigma X_{\text{3}}^{g_{\text{13}}}+K_{\text{13}}^{}}{X_{\text{3}}^{g_{\text{13}}}+K_{\text{13}}^{}}\right)-\beta_{\text{1}}X_{\text{1}},\;$$

(5) $$\frac{\text{d}X_{\text{2}}}{\text{d}t}=\alpha_{\text{2}}X_{\text{1}}X_{\text{5}}{\left(\frac{X_{\text{3}}}{X_{\text{3}}+K_{\text{23}}}\right)}^{n}-\beta_{\text{2}}X_{\text{2}},\;$$

(6) $$\frac{\text{d}X_{\text{3}}}{\text{d}t}=\alpha_{\text{31}}X_{\text{2}}X_{\text{6}}+\alpha_{\text{32}}X_{\text{7}}-\beta_{\text{3}}X_{\text{8}}{\left(\frac{X_{\text{3}}}{X_{\text{3}}+K_{\text{23}}}\right)}^{m}.$$

*X*_1_ represents the concentration of mRNA that encodes some critical enzyme of the biosynthetic pathway. Transcription is dependent on the cognate amino acid concentration, *X*_3_, and is described by a rational function with a Hill number of *g*_13_, and a ratio between minimum and maximum rates $\sigma =V_{\text{1L}}/V_{\text{1H}}$. The loss of *X*_1_ is dominated by first-order degradation of mRNA. *X*_2_ represents the concentration of the critical enzyme, which is assumed to be stable. As was shown in the previous section, the rate of translation, or protein production, is drastically affected by the limiting amino acid concentration, *X*_3_, and the exponent *n* is the number of cognate amino acids in the critical enzyme. The loss of *X*_2_ is dominated by first-order dilution in an exponentially growing cell, and therefore *β*_2_ is equal to the growth rate constant, which is in turn affected by the availability of free amino acid, or $\beta_{\text{2}}=\mu =\mu_{\text{M}}{\left[X_{\text{3}}/(X_{\text{3}}+K_{\text{23}})\right]}^{m}$, where *μ*_M_ is the maximum growth rate constant when *X*_3_ is in excess. The free cognate amino acid concentration, *X*_3_, can be increased by biosynthesis or import from an external supply, each represented by a positive term. The free cognate amino acid pool is depleted by the cellular demand for amino acid, which we assume is dominated by protein synthesis. If the amino acids are quickly recycled from the aborted ribosome–peptide complex, then intuitively the rate of amino acid utilization is the rate of successful protein production, and the final term of equation (6) is therefore similar to the first term of equation (5). We assume that the exponent *m* is the average number of cognate amino acids in each protein of the expressed proteome, rather than *n*, the average number in the critical enzyme of the corresponding biosynthetic pathway. Furthermore, if the cell produces *P* proteins, each with an average number of cognate amino acids *m*, at a rate equal to that of the critical enzyme in the biosynthetic pathway, then $\beta_{\text{3}}=Pm\alpha_{\text{2}}X_{\text{5}}$.

To simplify the analysis, and without loss of generality, the variables and parameters are normalized with respect to initial concentrations and a chosen time constant: $x_{i}=X_{i}/X_{i\text{0}}$, $k_{i}=K_{i}/X_{\text{30}}$, and $\tau =t\mu_{\text{M}}$. The normalized system is described by equations (7–9):

(7) $$\frac{\text{d}x_{\text{1}}}{\text{d}\tau }=A\left[\left(\frac{1+k_{\text{13}}}{\sigma +k_{\text{13}}}\right)\left(\frac{\sigma x_{\text{3}}^{g_{\text{13}}}+k_{\text{13}}}{x_{\text{3}}^{g_{\text{13}}}+k_{\text{13}}}\right)-x_{\text{1}}\right],\;$$

(8) $$\frac{\text{d}x_{\text{2}}}{\text{d}\tau }=\left[x_{\text{1}}{\left(1+k_{\text{23}}\right)}^{n}{\left(\frac{x_{\text{3}}}{x_{\text{3}}+k_{\text{23}}}\right)}^{n}-x_{\text{2}}{\left(1+k_{\text{23}}\right)}^{m}{\left(\frac{x_{\text{3}}}{x_{\text{3}}+k_{\text{23}}}\right)}^{m}\right],$$

(9) $$\frac{\text{d}x_{\text{3}}}{\text{d}\tau }=B\left[Cx_{\text{2}}+(1-C)x_{\text{7}}-{\left(1+k_{\text{23}}\right)}^{m}{\left(\frac{x_{\text{3}}}{x_{\text{3}}+k_{\text{23}}}\right)}^{m}\right],\;$$

where $A=\frac{\beta_{\text{1}}}{\mu_{\text{M}}}$, $B=\frac{\beta_{\text{3}}X_{\text{80}}}{\mu_{\text{M}}X_{\text{30}}}$, and $C=\frac{\alpha_{\text{31}}X_{\text{60}}X_{\text{20}}}{\alpha_{\text{31}}X_{\text{60}}X_{\text{20}}+\alpha_{\text{32}}X_{\text{70}}}$.

It should be noted that care is taken when normalizing the system. To create a well-controlled comparison between systems with different parameters, the terms were chosen to ensure that the corresponding reaction rates in each system were equal at the initial conditions. Simple inspection confirms that the gain and loss of each species at the initial conditions is unity, no matter what the parameter values.

### Parameter Estimation

The mathematical model described by equations (7–9) contains nine parameters: *A*, *B*, *C*, *g*_13_, *σ*, *k*_13_, *k*_23_, *m*, and *n*. Of the nine, five are aggregates of other parameters. Table 1 lists the estimated parameter values. Where possible, the parameters are estimated based on published data for *E. coli*. In the remaining cases, reasonable estimates are made to reflect expected operating conditions. The aggregate parameter *A* is calculated based on published values. Similarly, *B* is based on published values and a reasonable estimate for *P*. *C* is chosen to reflect a heavily repressed biosynthetic pathway during growth in an initial state of amino acid abundance. *k*_13_ and *k*_23_ represent the critical thresholds of transcriptional and translational regulation, respectively, and are normalized relative to the initial cognate amino acid concentration, *X*_30_. Their values are chosen so that the initial rate of transcription, based on *X*_30_, is near minimum but still within the regulatory regime, whereas the initial rate of global translation, also based on *X*_30_, is near maximum but still within the regulatory regime.

**Table 1.** Parameters Estimates for Amino Acid Biosynthesis.

Parameter	Description	Value Used	Sources
*β*_1_	Rate constant of mRNA degradation	1 min^−1^	Leive and Kollin (1967); Blundell and Kennell (1974)
*μ*_M_	Rate constant of maximum growth and enzyme dilution	0.01 min^−1^^a^	Bremer and Dennis (1996)
*α*_2_*X*_5_	Rate of initiation of translation	20 min^−1^^b^	Draper (1996)
*X*_30_	Initial concentration of free cognate amino acid	75 µM	Sundararaj et al. (2004)
*X*_80_	Initial concentration of total mRNA	1 µM	Sundararaj et al. (2004)
*A*	Normalized rate constant of mRNA degradation	100	
*B*	Normalized initial maximum supply of cognate amino acid	10^6^	
*C*	Initial fraction of cognate amino acid autosynthesized	0.01	
*g*_13_	Strength of repression	2	
*σ*	Potential capacity (inverse)	10^−4^^c^	
*k*_13_	Concentration for half-maximal velocity raised to the power *g*_13_ (*K*_13_ relative to $X_{30}^{g_{13}}$)	0.001	
*k*_23_	Concentration for half-maximal velocity through step *i* (*K*_23_ relative to *X*_30_)	0.1	
*m*	Average number of cognate amino acids in a protein	16^d^	Tatusova et al. (1999); Rudd (2000); Pruitt et al. (2005)

Note.—The parameter values are estimated based on published data for *Escherichia coli*, or reasonable estimates are made to reflect expected operating conditions.

^a^Rate of dilution dominates rate of degradation; slowest growth rate as observed on minimal media.

^b^Rate at which efficient mRNAs initiate translation of trp.

^c^Potential capacity may not be observed capacity.

^d^Based on average protein length and an amino acid composition of 0.05.

### Dynamic Response to Starvation

**Fig. 4.**
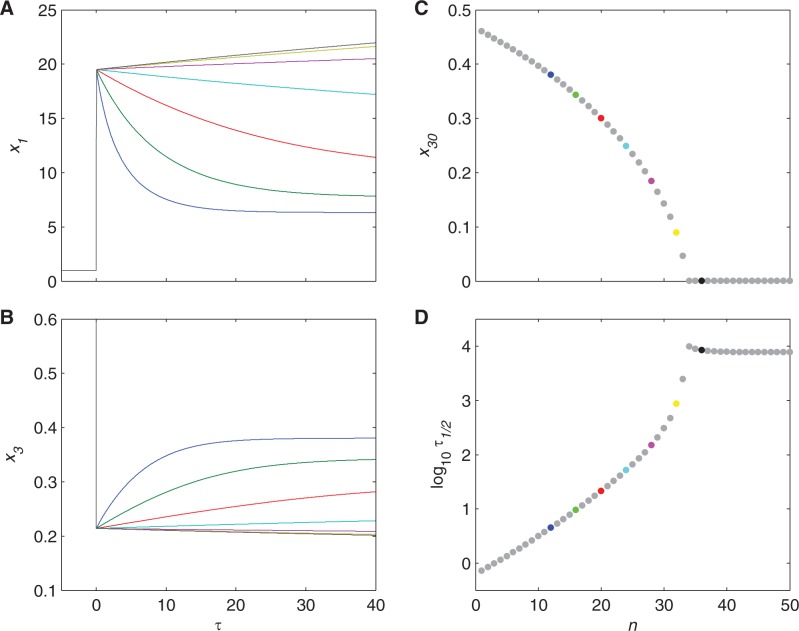
Dynamics of cognate amino acid concentration during starvation. (*A*) Normalized concentration of mRNA for the critical enzyme, *x*_1_, and (*B*) normalized concentration of free cognate amino acid, *x*_3_, during complete and instantaneous starvation. The time scale is normalized to the rate of dilution in the growing cell, or $\tau =t\mu_{\text{M}}$. (*C*) Final concentration of free cognate amino acid, *x*_30_, when the simulation continues to steady state, and (*D*) the speed of the response after the initial rapid drop, or the time *τ*_1/2_ required to cover half the distance to the final steady-state concentration. The number of cognate amino acids in the critical enzyme of the biosynthetic pathway varies from 1 to 50; specific values are *n* = 12 (blue), 16 (green), 20 (red), 24 (light blue), 28 (pink), 32 (yellow), and 36 (black). The average number of cognate amino acids in the proteins of the proteome, *m*, is 16. Values of *n* < *m* represent a low cognate bias in the biosynthetic pathway, whereas values of *n* > *m* represent a high cognate bias.

We define the cognate bias as *n* − *m*, or the difference between the number of cognate amino acids in a critical enzyme of the corresponding biosynthetic pathway, *n*, and the average number in the proteome, *m*. If the critical enzyme of the pathway is compositionally similar to the rest of the proteome, then there is no bias, or *n* − *m* = 0. Low bias represents the case where there are relatively fewer cognate amino acids in the biosynthetic pathway, or *n* − *m* < 0, whereas high bias represents the case where there are more, or *n* − *m* > 0. To examine the effect of cognate bias, various systems with different biases were computationally simulated in response to rapid and complete starvation: The normalized external supply of amino acid, *x*_7_, was decreased from 1 to 0 at *τ* = 0. The results are shown in figure 4. All of the systems immediately experience a rapid drop in free cognate amino acid and a commensurate rise in the mRNA that encodes the biosynthetic pathway. The response is expected, as the large cellular demand quickly depletes the free amino acid reserves. Systems with low (blue) or no (green) cognate bias compensate by derepression and stabilize at a new steady-state amino acid concentration. Systems with a slight high bias (red and light blue) recover more slowly, but eventually stabilize as well (see the final values in fig. 4*C*). Systems with slightly higher bias (pink and yellow) do not recover, but do stabilize at lower amino acid concentrations. Figure 4*C* and *D* clearly shows how the final steady-state concentration and response times vary with bias: The higher the bias, the slower the response and the lower the final steady-state concentration of free amino acid. Similar behavior is observed when the parameter values are varied—*A* between 5 and 100, *B* between 10^4^ and 10^6^, and *m* between 4 and 16—indicating that the result is insensitive to the values of the parameters. Furthermore, the behavior changes significantly between *n* = 32 (yellow) and 36 (black), at which point the amino acid concentration does not appear to stabilize at all, but rather continues on a trajectory toward zero, suggesting the full failure of the system. However, the response time is extremely slow, and the large exponents magnify rounding errors, which may introduce uncertainty in the final steady-state values. Nevertheless, the concentrations do eventually reach zero, which is confirmed by the following analysis of the steady states.

### Steady-State Concentrations after Starvation

At steady state, the derivatives that represent the changing concentrations vanish in equations (7–9). Also, complete starvation sends the external amino acid supply *x*_7_ to zero. Simple inspection of the equations when d*x_i_*/d*τ* and *x*_7_ are zero reveals that *x*_3_ = 0 is always a solution, meaning that a steady-state amino acid concentration of zero is a possibility. Subsequent manipulation of equations (7–9) yields the following equation, in terms of the free amino acid concentration *x*_3_ and the estimated parameters of the system:

(10) $$C\left(\frac{1+k_{13}}{\sigma +k_{13}}\right)\left(\frac{\sigma x_{3}^{g_{13}}+k_{13}}{x_{3}^{g_{13}}+k_{13}}\right)={\left(1+k_{23}\right)}^{2m-n}{\left(\frac{x_{3}}{x_{3}+k_{23}}\right)}^{2m-n}$$.

**Fig. 5.**
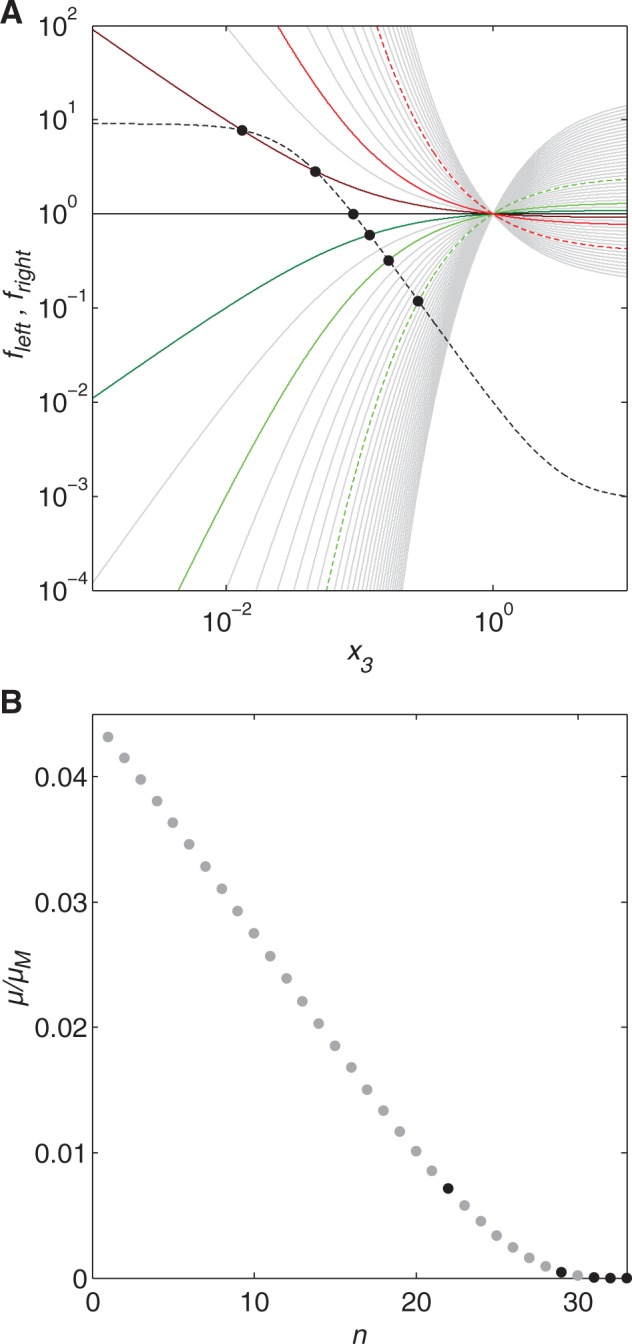
Steady-state cognate amino acid concentration and growth rate after starvation. (*A*) After starvation (*x*_7_ = 0), the normalized, steady-state value of free amino acid concentration *x*_3_ is found at the intersection of *f*_left_, the value of the left side of equation (10) (dotted black) and *f*_right_, the value of the right side of equation (10) for *n* = 1–50 (gray), specifically *n* = 22 (dashed green), 29 (green), 31 (dark green), 32 (black), 33 (dark red), 35 (red), and 42 (dashed red). The average number of cognate amino acids in the proteins of the proteome, *m*, is 16. Cases where *n < 2m* (dashed green, green, and dark green) have a single stable, positive steady state, and are considered safe. Cases where 2*m < n < m_c_* (dark red) have multiple potential outcomes. Cases where *n > m_c_* (red and dashed red) have only one steady state at zero (not evident on the logarithmic axis), and are therefore fatal. (*B*) Normalized growth rate *μ*/*μ*_M_ = [*x*_3_/(*x*_3_ + *k*_23_)]^*m*^ given the steady-state amino acid concentrations found for *n* = 1–33 (gray), specifically for *n* = 22, 29, 31, 32, and the coincident doublet at 33 (black).

There is no closed-form solution for *x*_3_ given the general form of equation (10). However, when both sides of the equation, *f*_left_ and *f*_right_, are plotted as shown in figure 5*A*, the intersections identify the values of *x*_3_, or the normalized steady-state concentrations, that satisfy equation (10). In figure 5*A*, *f*_left_ is drawn for *g*_13_ = 2, whereas *f*_right_ is drawn for several different values of cognate bias, or *n* − *m*. Overall, there are six labeled intersections in figure 5*A*, and a numerical solver was used to verify and refine each of them, producing six potential steady-state concentrations for *x*_3_: 0.013, 0.047, 0.090, 0.12, 0.17, and 0.28. However, the number of intersections is not equal to the number of curves—for some values of bias, *f*_left_ and *f*_right_ clearly intersect at a single point; for other values, the curves intersect at two points or not at all.

Figure 5*A* reveals three distinct cases of interest. When n≤2m, the system has only one intersection, and therefore one steady state, in addition to the zero steady state that was previously identified. The eigenvalues of the system linearized near the steady states confirm, as the simulations indicated, that the positive steady state is stable whereas the steady state at zero is unstable. This implies only one possible outcome for n≤2m: A low, albeit nonzero, stable steady-state amino acid concentration, which is considered safe. When *n* is slightly greater than 2*m*, but still below some threshold *m_c_*, the system has two intersections in figure 5*A*, or two nonzero steady states, in addition to the ever-present steady state at zero. The eigenvalues of the system linearized near these steady states confirm, as the simulations indicated, that the high steady state is stable, the intermediate steady state is unstable, and the zero steady state is stable. Thus, there are two possible outcomes: Recovery or full system failure. When *n* is much greater than 2*m*, or greater than the threshold *m_c_*, there are no intersections in figure 5*A*, but the steady state at zero is still stable, implying that the only possible outcome is full failure. In this final case, the high cognate bias is fatal. Indeed, the steady-state analysis confirms the results indicated by the dynamic simulations. The key determinant of survival is the cognate bias, or the relative values of *n* and *m*. In particular, the key measure is *n* − 2*m*, or the “critical bias.” Values of n−2m≤0 are safe, whereas values of *n* − 2*m* > 0 are potentially fatal. Furthermore, figure 5*B* shows that even when *n* < *m_c_*, and the system therefore stabilizes at a nonzero steady state amino acid concentration, the normalized growth rate *μ*/*μ*_M_ = [*x*_3_/(*x*_3_ + *k*_23_)]^*m*^ varies with *n*, and the lower the bias, the higher the growth rate.

### Statistical Evidence of Bias across Genomes

A biological design that would protect against full failure during starvation would be a low cognate bias for the critical enzymes of the amino acid biosynthetic pathways. Past work suggests that there is statistical evidence of low cognate bias in the pathways of some organisms, including *B. subtilis*, *E. coli*, and *S. enterica* (serovar Typhimurium) (Alves and Savageau 2005). However, that analysis used a different model and a different measure of bias based on relative percentage of amino acid composition. Our model presented here indicates that the key measure of bias should instead be based on relative number of cognate amino acids. Furthermore, there is now a substantially larger body of genomic data to mine.

To search for evidence of cognate bias in the genomes of sequenced prokaryotes, we utilized the MetaCyc pathway database (Caspi et al. 2011) and the UniProt protein database (UniProt Consortium 2014). In our model, *n* represents the number of cognate amino acids in some critical enzyme of the amino acid biosynthetic pathway. However, the identification of a critical enzyme is problematic. It could be a rate-limiting enzyme—and yet different enzymes may be rate limiting under different conditions. The selection could be based on other factors, including activity, half-life, normal concentration, or regulative capacity, each of which is relatively uncharacterized when compared with the wealth of sequence data. Furthermore, amino acid biosynthetic pathways are intertwined, which complicates the identification of a critical enzyme for a specific biosynthetic pathway. Nevertheless, it can be argued that the selective pressure for cognate amino acid bias applies to all of the regulated enzymes of the pathway—after all, the entire pathway must be upregulated in response to starvation—and the last enzyme in each pathway can be uniquely and easily identified. Using MetaCyc, we compiled a list of the last enzymes in all of the known pathways leading to one of the 20 fundamental amino acids, shown in supplementary table S1, Supplementary Material online. From UniProt, we downloaded the complete proteomes of 1,816 completely sequenced prokaryotes. Within each proteome, we searched for the biosynthetic enzymes identified in supplementary table S1, Supplementary Material online. If an enzyme was found, we used the number of cognate amino acids in the enzyme to represent *n* in our model. If more than one enzyme was found for the production of a particular amino acid, we assumed that the organism has multiple alternative pathways and the pathway with the lowest number of cognate amino acids is the most resilient; therefore the lowest of the numbers was used to represent *n*. Finally, we counted the number of cognate amino acids in each protein of the proteome and used the average number to represent *m*. The cognate bias, as we have shown, is *n* − *m*, and the critical bias is *n* − 2*m*. The resulting data, used in subsequent analyses, are included in supplementary table S2, Supplementary Material online.

**Fig. 6.**
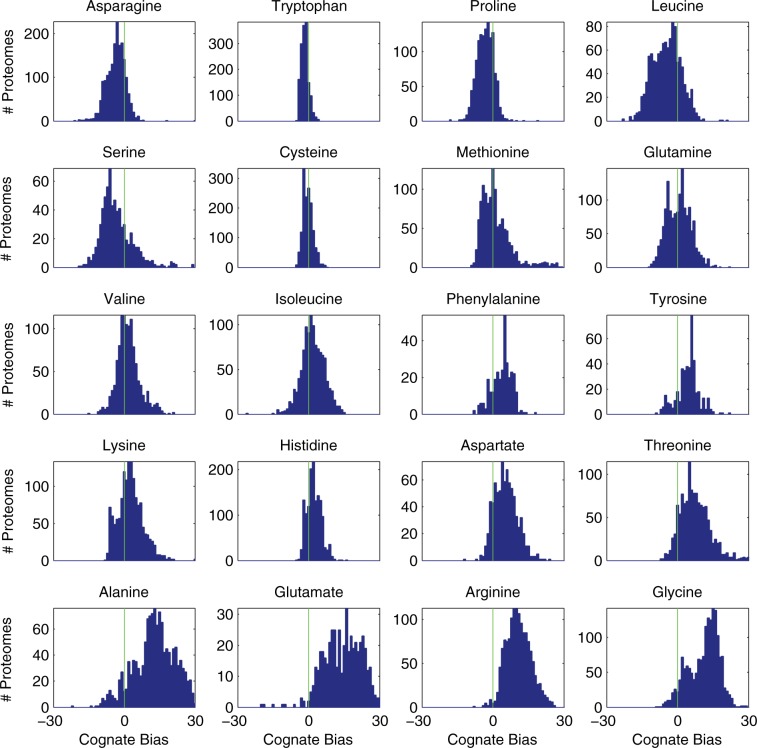
Histograms of cognate bias measured in complete proteomes. Each histogram describes the distribution of cognate biases across the proteomes of fully sequenced prokaryotes in the UniProt database. Here, the cognate bias is the difference between the number of cognate amino acids in the final enzyme of the biosynthetic pathway *n* (or the minimum *n* if there are multiple pathways) and the average number of cognate amino acids in each protein of the putative proteome *m*. A low bias is less than zero (*n* − *m* < 0), whereas a high bias is greater than zero (*n* − *m* > 0). With the exception of Alanine and Glutamate, only 19 points fall outside the depicted range; the full ranges for Alanine and Glutamate are shown in supplementary figure S1, Supplementary Material online.

Figure 6 displays histograms of the cognate biases measured for each of the 20 amino acids over all 1,816 proteomes. As expected, histograms of the critical biases are similar in shape and shown in supplementary figure S2, Supplementary Material online. There are obvious cases of extreme cognate bias in figure 6: Tryptophan, in almost every case, has a low bias, whereas Arginine, in almost every case, has a high bias. To statistically analyze the significance of the biases, we performed a sign test (see Materials and Methods), a nonparametric test that does not assume a particular population distribution and measures the probability that the values are drawn from a population with a median value of zero, or no bias. The results of the test are listed in table 2. Low *P* values indicate that the population is biased, and the sample median indicates whether it is biased high or low. The results indicate that there is significant statistical evidence of low cognate bias (*P* < 0.001) in the biosynthetic pathways of six amino acids: Asparagine, Tryptophan, Proline, Leucine, Serine, and Cysteine. All but one of the pathways—Glutamate—show statistically significant evidence of low, or safe, critical bias. Organisms that have a close phylogenetic relationship might be expected to have similar biases and might not be considered independent samples. However, we obtained essentially the same results for the UniProt reference proteomes, which have been selected specifically to provide a wide phylogenetic distribution (UniProt Consortium 2014). Moreover, when clustering on the basis of cognate bias, even organisms that have a close phylogenetic relationship split into different clusters, as shown in the following section.

**Table 2.** Statistical Measures of Bias.

Amino Acid	Number of Organisms	Median Cognate Bias	*P* Value	Median Critical Bias	*P* Value
Asparagine	1,790	−3.1	1e-166	−15.7	0
Tryptophan	1,526	−1.7	6e-161	−5.4	0
Proline	1,288	−3.0	3e-112	−16.6	5e-281
Leucine	1,142	−4.4	2e-80	−36.3	2e-249
Serine	694	−4.5	6e-40	−23.0	1e-142
Cysteine	1,509	−0.5	8e-12	−3.3	8e-246
Methionine	1,229	0.1	0.2	−7.9	3e-141
Glutamine	1,443	0.7	0.0005	−10.3	1e-273
Valine	1,142	1.3	1e-17	−20.7	3e-250
Isoleucine	1,142	1.6	2e-23	−18.0	7e-246
Phenylalanine	315	4.6	3e-29	−7.4	9e-68
Tyrosine	433	5.1	3e-46	−3.7	1e-55
Lysine	1,470	2.3	8e-52	−13.4	1e-307
Histidine	1,409	2.1	6e-91	−4.2	5e-209
Aspartate	799	5.2	5e-99	−11.7	2e-165
Threonine	1,253	6.6	9e-181	−9.7	7e-188
Alanine	1,427	13.8	1e-216	−12.7	7e-204
Glutamate	1,046	45.0	7e-220	25.4	3e-23
Arginine	1,438	10.5	1e-295	−7.2	3e-166
Glycine	1,727	12.6	3e-303	−10.1	4e-264

Note.—For each amino acid, the table lists the number of organisms in which a biosynthetic pathway enzyme was found, the median cognate bias and critical bias of the population, and their *P* values based on a sign test (see Materials and Methods). Low *P* values (*P* < 0.001) indicate a statistically significant high or low (positive or negative) bias.

### Clustering of Cognate Bias Compared with Taxonomy

**Fig. 7.**
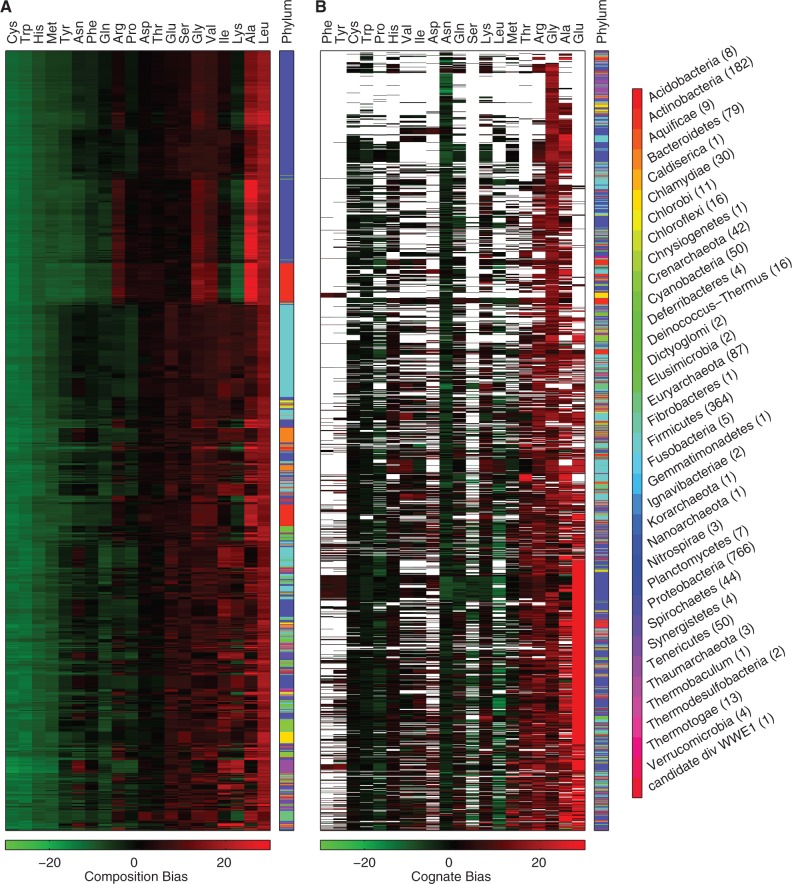
Hierarchical clustering of composition bias and cognate bias profiles, based on bias similarity in the complete proteomes of the UniProt database. (*A*) The clustering is based on the composition bias: The average difference between the number of an amino acid in each protein and the expected number, or 5% of the protein length. High composition bias (red) indicates that the amino acid is relatively overrepresented in the proteins of the proteome; low bias (green) indicates that the amino acid is relatively underrepresented. Each row describes the biases measured for a sequenced organism, and the vertical ordering is based on the similarity of the bias profile across all 20 amino acids, with rows closer to the top being more similar. Each column represents one of the 20 amino acids, and the horizontal ordering is based on the similarity of the bias values for that given amino acid over all of the organisms, with columns to the left being more similar. The phylum of each row, or organism, is plotted in a distinct color to the right. The number of organisms in each phylum is shown in parenthesis in the legend. (*B*) The clustering is based on the cognate bias: The difference between the average number of a given amino acid in each protein of the proteome and the number in the final enzyme of the amino acid biosynthetic pathway. A high cognate bias (red) represents a higher number in the pathway; a low bias (green) represents a lower number in the pathway. Missing values (white) indicate that the biosynthetic pathway was not found in the proteome. The vertical and horizontal ordering are based on similarity, as described in (*A*). The phylum of each row, or organism, is plotted to the right as in (*A*).

Without any prior knowledge, each protein of the proteome would naively be expected to contain equal amounts, or 5%, of each amino acid. However, it is widely know that this is not the case, and figure 7*A* depicts our calculation of the amino acid composition biases for each of the completely sequenced prokaryotes found in the UniProt database. The composition bias is measured as the average difference between the number of an amino acid in each protein of the proteome and the expected number, or 5% of the length of the protein. A positive number, or high composition bias, indicates a higher than expected number of a given amino acid in the proteins of the proteome; a low bias indicates a lower than expected number. Figure 7*A* shows that Cysteine, Tryptophan, Histidine, and Methionine are consistently underrepresented in the proteomes, whereas Leucine is consistently overrepresented. The remaining amino acids vary between a high and low composition bias, depending on the organism. The vertical ordering is sorted by the similarity of the organisms across all 20 amino acids, and reveals large and small clusters with similar bias profiles. The adjacent bar indicates the taxonomic phylum of each organism, and in several cases the bias clusters roughly correspond to phyla, especially in the highly represented cases of Proteobacteria, Fimicutes, and Actinobacteria.

On the other hand, figure 7*B* depicts the measured cognate biases for each of the complete proteomes. A positive number, or high cognate bias, indicates a higher than expected number of a given amino acid in the final enzyme of the biosynthetic pathway; a low bias indicates a lower than expected number. Missing values indicate that none of the known enzymes, and presumably none of the known pathways, was found in the proteome. Note that the cognate bias, by definition, is measured with respect to the composition bias, and so it is remarkable, for example, that Tryptophan, which is already underrepresented in the proteome, tends to have an even lower number in the biosynthetic pathway. The vertical ordering of the cognate bias profiles, based on similarity, reveals fewer obvious clusters than the ordering based on composition bias, although a small cluster in the center includes *E. coli* and other organisms that can synthesize all 20 amino acids, and a larger cluster at the bottom includes proteomes with a very high Glutamate cognate bias. Likewise, clustering based on cognate bias does not appear to correlate as strongly with the taxonomic phyla as does the clustering based on composition bias, especially in the highly represented cases of Proteobacteria, Fimicutes, and Actinobacteria. A similar result is apparent when the proteomes and pathways are clustered by critical bias, as shown in supplementary figure S3, Supplementary Material online. (Raw data for fig. 7A and B, and supplementary fig. S3, can be found in supplementary tables S3–S5, respectively.) Indeed, the larger clusters apparent in figure 7*A* are broken up and scattered in figure 7*B* and supplementary figure S3, suggesting that any similarity in cognate bias may depend less on taxonomy and more on the environment.

## Discussion

Part of the cell’s stringent response to starvation is the upregulation of amino acid biosynthetic pathways, and yet the stringent response might not be enough to protect the cell from the shock of rapid starvation. Cognate bias—relatively fewer cognate amino acids in the corresponding pathway synthesizing the amino acid—could avoid a potential catch-22, in which the emergency response requires the very amino acid that has disappeared from the environment. Past work (Alves and Savageau 2005) suggested that there is a low cognate bias in the pathways of some amino acids, that the bias tends to be lower for the key enzymes in the pathway, and that the profile of bias differs between *E. coli, S. enterica* (serovar Typhimurium), and *B. subtillis*—organisms from different environmental niches. However, the analysis used a different model and a different measure of bias based on relative percentage of amino acid composition, whereas our more detailed model indicates that the key measure of bias should be based on the relative number of cognate amino acids. To illustrate the difference, consider three proteins of 100, 200, and 400 amino acids, each containing two cognate amino acids. The first consists of 2% cognate amino acid, the second consists of 1%, and the third 0.5%. Based on relative percentages, the first protein has the highest bias, whereas the third has the lowest bias; but based on relative numbers of cognate amino acids, their bias is the same. The impact of the different definitions can be significant.

Dynamic simulations and a steady-state analysis of our model show that important aspects of the system depend on the cognate bias, the number of cognate amino acids in the corresponding biosynthetic pathway (*n*) relative to the number in the expressed proteome (*m*), or *n*-*m*. The lower the cognate bias, the faster the system responds to starvation and the higher the recovered concentration of the free amino acid pool will be, potentially creating a selective evolutionary pressure to lower the cognate bias. Furthermore, our results confirm that a key determinant of the cell fate is the cognate bias. The crucial measure, based on cognate bias, is the critical bias, or *n* − 2*m*. A low critical bias is always safe, whereas a very high critical bias is fatal. An ambiguous critical bias—slightly high, but below some threshold—can lead to either recovery or failure, depending on the initial conditions and the dynamics of the system. The results suggest immediate predictions that can be experimentally tested. Although single amino acid starvation experiments have been performed in bacteria over the years (Venetianer 1969; Stephens et al. 1975; Shand et al. 1989; Parish 2003; Ohashi et al. 2008), they have not comprehensively tested all of the amino acids. We propose that samples taken from an unstressed culture, grown on rich medium, in steady-state exponential growth, could be used to inoculate a series of cultures grown on 20 different chemically defined media, where each medium has an excess of all amino acids except one. We predict new cultures that upregulate pathways with low critical bias will recover, whereas those that upregulate pathways with high critical bias may fail, or at least experience a long lag in recovery. Furthermore, the model is general enough to make predictions for a variety of organisms. Measures of critical bias should predict relative growth rates and differential recovery times between two organisms starving for the same amino acid or the same organism starving for different amino acids.

A key aspect of our analysis is the changing environment. In a broader context, three classes of amino acids likely correspond to different environmental effects on the proteome: For amino acids that are never required by the organism, the biosynthetic pathway can be dispensed with entirely. For amino acids that are always required by the organism, regulation can be dispensed with, and the amino acids synthesized constitutively without regard for cognate bias. For all other environments, regulation would be advantageous, and a compensating cognate bias would likely exist for amino acids that experience the most frequent and extreme fluctuations in the organism’s natural environment. In figure 7*B*, the missing data points may indicate biosynthetic pathways the organisms have eliminated. High bias may represent the second case: Amino acids that are constitutively produced without regard to bias because they are absent in the environment. In this case, the evidence might suggest that other mechanisms lead to the selection for high cognate bias. Our analysis and results shed light on the last case, a dynamic environment where the amino acid supply is in flux.

An analysis of the proteomes for over 1,800 fully sequenced organisms indicates a statistically low cognate bias in six amino acid biosynthetic pathways, and a low critical bias in all but one. The results open up the possibility that an organism’s genome may reflect the nutritional stress that the organism naturally encounters. It should be noted that large protein data sets such as UniProt are built in part by homology with known proteins, which could skew the results toward a few well-studied organisms such as *E. coli*. On the other hand, we obtained similar results when analyzing only the reference proteomes in UniProt, which represent a small, diverse set of well-studied model organisms. Furthermore, we have only considered the case of immediate and complete starvation, whereas organisms naturally experience a variety of transitions from abundance to scarcity and vice versa, sometimes in patterns. For example, *E. coli* encounters periods of starvation and abundance while passing through the guts of successive hosts (Savageau 1983). Future work could explore a full range of environmental conditions and patterns of stress. Ideally, transcriptomic, proteomic, and metabolomic studies would be performed over a full range of concentrations for a single amino acid. In fact, genome-wide studies continue to advance our understanding of the stringent response in *E. coli* (DeRisi et al. 1997; Chang et al. 2002; Durfee et al. 2008; Traxler et al. 2008; Jozefczuk et al. 2010) as well as other organisms (Brockmann-Gretza and Kalinowski 2006; Vercruysse et al. 2011; Gaca et al. 2012), but the studies have not focused on individual amino acid starvation.

The stringent response is a drastic and complex measure taken to cope with a changing environment. Likewise determining the natural environment of most organisms is a challenging task. Our model of starvation and the stringent response establishes a link between the genome and the environment. The results suggest at least one design principle—a low cognate bias—that cells may use to cope with nutritional stress. Armed with that knowledge, we may be able to mine our growing genomic storehouse for clues to an organism’s environmental niche.

## Materials and Methods

All analyses were performed with custom scripts and the built-in functions of MATLAB R2009a (7.8). The dynamic simulations utilized the built-in MATLAB solver for stiff ODEs: ode15s. The steady-state solutions were found by sampling equation (10), and then verified and polished using the built-in MATLAB function fzero. The linearization of the system and the evaluation of the local stability of the steady states were accomplished with the aid of the MATLAB Symbolic Math Toolbox.

Information on amino acid biosynthetic pathways and their final enzymes was curated manually from the MetaCyc pathway database (Caspi et al. 2011). Protein data were retrieved, parsed, and analyzed using custom MATLAB scripts from the UniProt protein database (UniProt Consortium 2014). The resulting data, used in the subsequent analyses, can be found in supplementary tables S1–S5, Supplementary Material online. The statistical tests for significant cognate bias and critical bias utilized the sign test, a nonparametric test that does not make assumptions about the population distribution, and measures the probability that values were drawn from a population with a median value of zero, or in this case, no bias. The sign test used here was one-sided, in order to measure whether the population deviated in a particular direction, either high or low. The sign tests were performed through the built-in MATLAB function signtest. The clustering analysis was based on nearest Euclidean distance and performed through the built-in MATLAB function linkage.

## Supplementary Material

Supplementary figures S1–S3 and tables S1–S5 are available at *Molecular Biology and Evolution* online (http://www.mbe.oxfordjournals.org/).

Supplementary Data
