# Supplementary material for: Evolution of a Genome-Encoded Bias in Amino Acid Biosynthetic Pathways Is a Potential Indicator of Amino Acid Dynamics in the Environment
Source: Mol Biol Evol. 2014 Aug 12;31(11):2865–78. doi: 10.1093/molbev/msu225 (PMC4209129; doi:10.1093/molbev/msu225)
Supplement: Supplementary Data [file supp_31_11_2865__index.html]

Evolution of a Genome-Encoded Bias in Amino Acid Biosynthetic Pathways Is a Potential Indicator of Amino Acid Dynamics in the Environment — Evolution of a Genome-Encoded Bias in Amino Acid Biosynthetic Pathways Is a Potential Indicator of Amino Acid Dynamics in the Environment — Supplementary Data 

# Evolution of a Genome-Encoded Bias in Amino Acid Biosynthetic Pathways Is a Potential Indicator of Amino Acid Dynamics in the Environment

## Supplementary Data

file

**Files in this Data Supplement:**

- Supplementary Data - pdf file
- Supplementary Data - xls file
- Supplementary Data - xls file
- Supplementary Data - xls file
- Supplementary Data - xls file
