# Supplementary material for: Evolution of a Genome-Encoded Bias in Amino Acid Biosynthetic Pathways Is a Potential Indicator of Amino Acid Dynamics in the Environment
Source: Mol Biol Evol. 2014 Aug 12;31(11):2865–78. doi: 10.1093/molbev/msu225 (PMC4209129; doi:10.1093/molbev/msu225)
Supplement: Supplementary Data [file supp_msu225_Supplementary_Information.pdf]

Evolution of a Genome Encoded Bias in Amino Acid Biosynthetic Pathways is a Potential Indicator of Amino Acid Dynamics in the Environment

Rick A. Fasani and Michael A. Savageau

Supplementary Information

Table of Contents

Supplementary Figure 1. Histograms of Cognate Bias for Alanine and Glutamate

Supplementary Figure 2. Histograms of Critical Bias for All Amino Acids

Supplementary Figure 3. Hierarchical Clustering of Critical Bias Profiles

Supplementary Table 1. Final Enzymes in Amino Acid Biosynthetic Pathways

Supplementary Table 2. Data for Computation of Bias by Organism and Pathway*

Supplementary Table 3. Data for Hierarchical Clustering of Composition Bias Profiles*

Supplementary Table 4. Data for Hierarchical Clustering of Cognate Bias Profiles*

Supplementary Table 5. Data for Hierarchical Clustering of Critical Bias Profiles*

*In a separate file

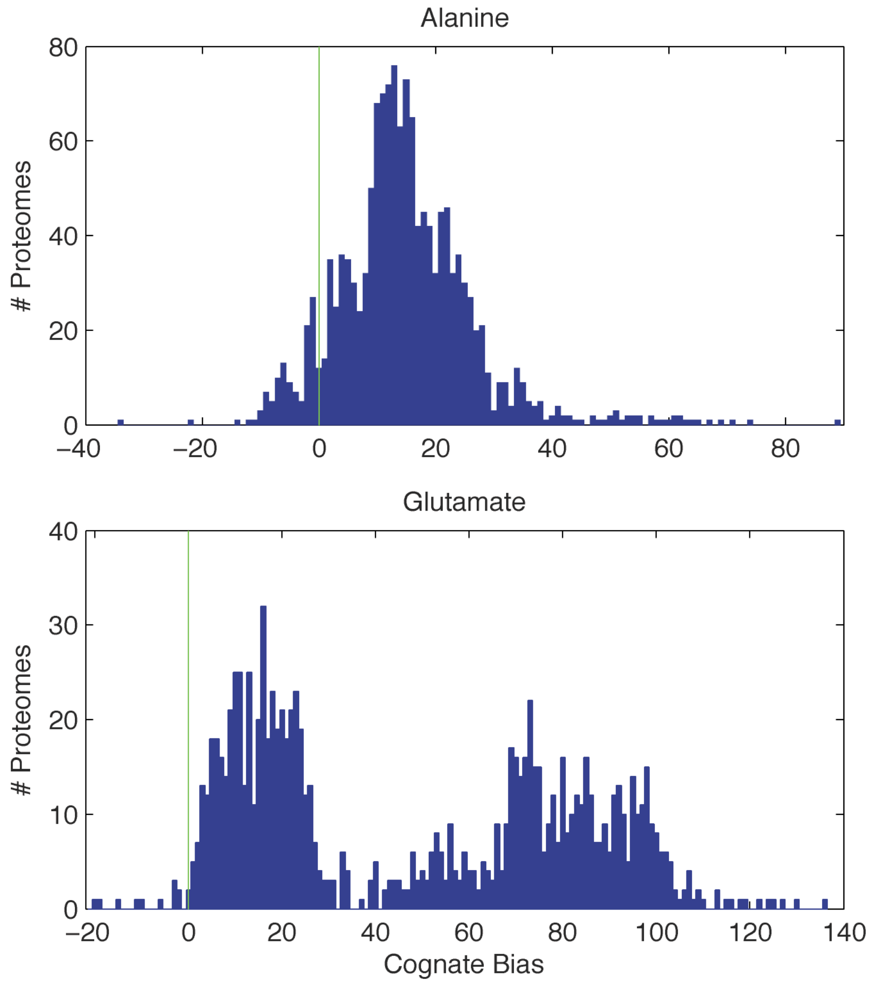

Supplementary Figure 1. Histograms of Cognate Bias for Alanine and Glutamate

Each histogram describes the distribution of cognate biases across the proteomes of fully sequenced prokaryotes in the UniProt database. Here, the cognate bias is the difference between the number of cognate amino acids in the final enzyme of the biosynthetic pathway n (or the minimum n if there are multiple pathways) and the average number of cognate amino acids in each protein of the putative proteome m. A low bias is less than zero ($n - m < 0$), while a high bias is greater than zero ($n - m > 0$).

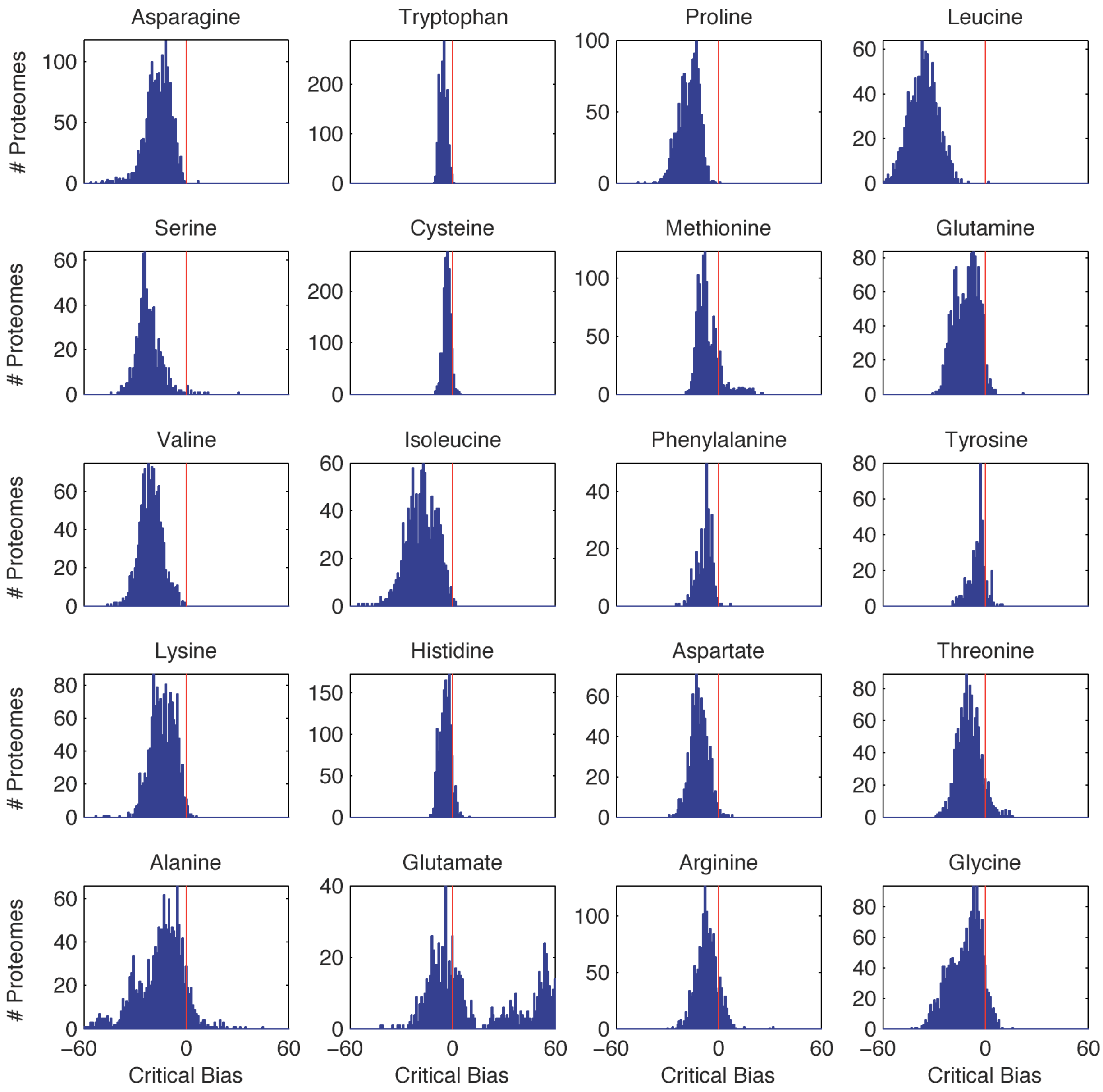

Supplementary Figure 2. Histograms of Critical Bias for All Amino Acids

Each histogram describes the distribution of critical biases ($n - 2m$). A critical bias less than zero ($n - 2m < 0$) is safe, because it has a single, positive, final steady-state concentration of free amino acid. On the other hand, a critical bias greater than zero ($n - 2m > 0$) is unsafe, because at least one potential fate—and in many cases the only fate—is a final steady-state concentration of zero.

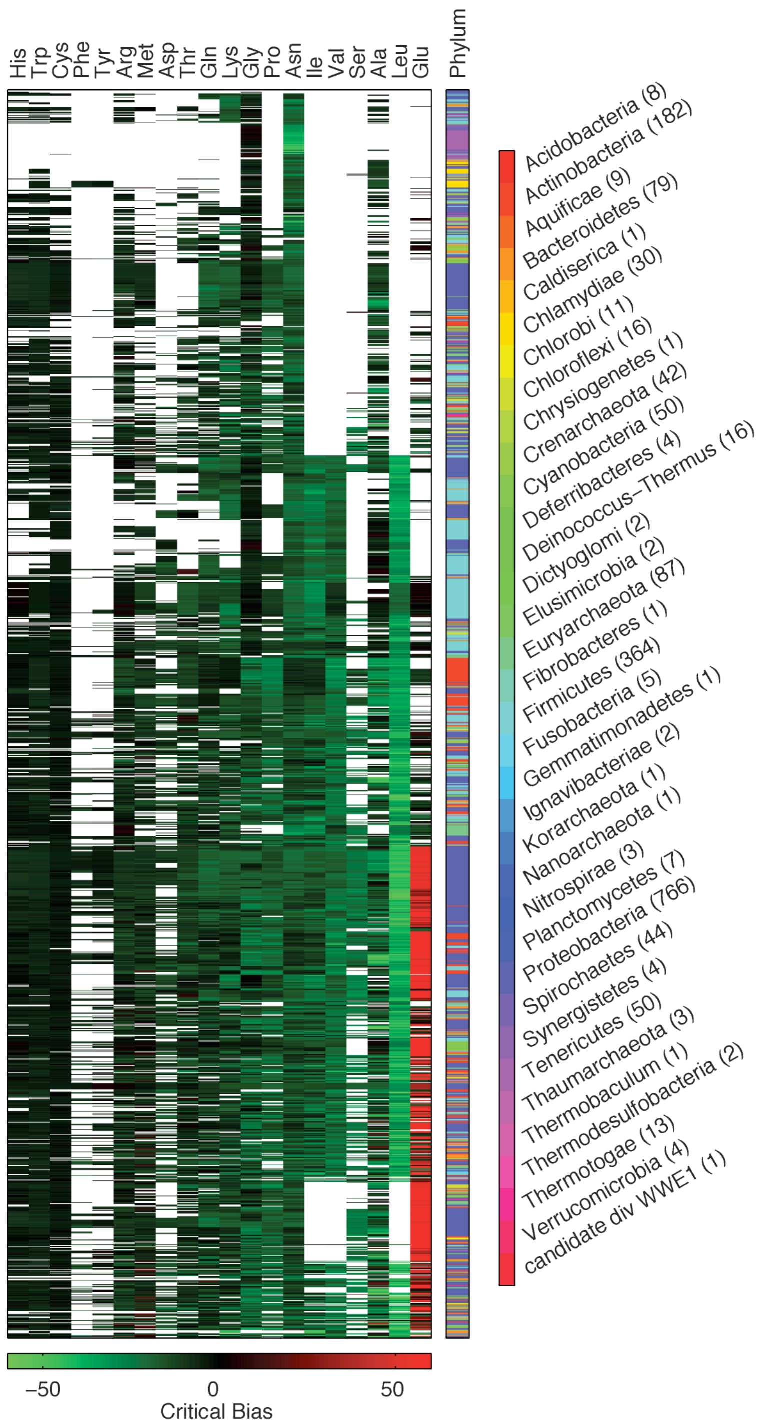

Supplementary Figure 3. Hierarchical Clustering of Critical Bias Profiles

Hierarchical ordering and clustering based on the critical bias ($n - 2m$) similarity in the complete proteomes of the UniProt database. A high critical bias (red) represents an unsafe bias; a low bias

(green) represents a safe bias. Missing values (white) indicate that the biosynthetic pathway was not found in the proteome. The vertical and horizontal ordering are based on similarity, as described in Figure 7. The phylum of each row, or organism, is plotted to the right as in Figure 7.

Supplementary Table 1. Final Enzymes in Amino Acid Biosynthetic Pathways

Amino Acid	MetaCyc Pathway	EC Number	Example Enzyme	Reaction
Alanine	alanine biosynthesis I	2.6.1.66	Valine--pyruvate transaminase	L-valine + pyruvate <=> 3-methyl-2-oxobutanoate + L-alanine
Alanine	alanine biosynthesis II	2.6.1.2	Alanine transaminase	L-alanine + 2-oxoglutarate <=> pyruvate + L-glutamate
Alanine	alanine biosynthesis III	2.8.1.7	Cysteine desulfurase	L-cysteine + acceptor <=> L-alanine + S-sulfanyl-acceptor
Arginine	arginine biosynthesis I; II (acetyl cycle); III; IV; citrulline-nitric oxide cycle	4.3.2.1	Argininosuccinate lyase	2-(N(omega)-L-arginino)succinate <=> fumarate + L-arginine
Asparagine	asparagine biosynthesis I	6.3.5.4	Asparagine synthase (glutamine-hydrolyzing)	ATP + L-aspartate + L-glutamine + H(2)O <=> AMP + diphosphate + L-asparagine + L-glutamate
Asparagine	asparagine biosynthesis II	6.3.1.1	Aspartate-ammonia ligase	L-aspartate + ammonia + ATP <=> L-asparagine + AMP + diphosphate
Asparagine	asparagine biosynthesis III (tRNA-dependent)	3.1.1.29	Aminoacyl-tRNA hydrolase	L-asparaginyl-tRNAasn + H2O -> L-asparagine + tRNAasn + 2 H+
Aspartate	aspartate biosynthesis; glutamate degradation II	2.6.1.1	Aspartate transaminase	L-aspartate + 2-oxoglutarate <=> L-glutamate + oxaloacetate
Cysteine	cysteine biosynthesis I	2.5.1.47	Cysteine synthase	O-acetyl-L-serine + hydrogen sulfide <=> L-cysteine + acetate + H+
Cysteine	cysteine biosynthesis II (RNA-dependent)	2.5.1.73	O-phospho-L-seryl-tRNA:Cys-tRNA synthase	O-phospho-L-seryl-tRNACys + hydrogen sulfide <=> L-cysteinyl-tRNAcys + phosphate + H+
Cysteine	cysteine biosynthesis III (mammalia); IV (fungi); cysteine biosynthesis/homocysteine degradation; homocysteine and cysteine interconversion	4.4.1.1	Cystathionine gamma-lyase	L-cystathionine + H(2)O <=> L-cysteine + NH(3) + 2-oxobutanoate
Glutamate	arginine degradation I (arginase pathway)	1.2.1.88*	1-pyrroline-5-carboxylate dehydrogenase	(S)-1-pyrroline-5-carboxylate + NAD(P)(+) + 2 H(2)O <=> L-glutamate + NAD(P)H
Glutamate	arginine degradation I (arginase pathway)	1.5.1.12*	1-pyrroline-5-carboxylate dehydrogenase	(S)-1-pyrroline-5-carboxylate + NAD(P)(+) + 2 H(2)O <=> L-glutamate + NAD(P)H
Glutamate	glutamate biosynthesis I	1.4.1.13	glutamate synthase	L-glutamine + 2-oxoglutarate + NADPH + H+ <=> 2 L-glutamate + NADP+

Glutamate	glutamate biosynthesis II	1.4.1.3	glutamate dehydrogenase 2	NAD(P)+ + L-glutamate + H2O <=> ammonia + NAD(P)H + 2-oxoglutarate + 2 H+
Glutamate	glutamate biosynthesis III	1.4.1.4	glutamate dehydrogenase	L-glutamate + NADP+ + H2O <=> ammonia + 2-oxoglutarate + NADPH + 2 H+
Glutamate	glutamate biosynthesis IV	1.4.1.14	glutamate synthase (NADH)	2 L-glutamate + NAD+ <=> L-glutamine + 2-oxoglutarate + NADH + H+
Glutamate	glutamate biosynthesis V	1.4.7.1	glutamate synthase (ferredoxin-dependent)	2 L-glutamate + 2 an oxidized ferredoxin <=> 2-oxoglutarate + L-glutamine + 2 a reduced ferredoxin + 2 H+
Glutamine	glutamine biosynthesis I; III	6.3.1.2	glutamine synthetase	ammonia + L-glutamate + ATP <=> L-glutamine + ADP + phosphate
Glutamine	L-glutamine biosynthesis II (tRNA-dependent)	6.3.5.7	glutamyl-tRNA(Gln) amidotransferase	L-glutamine + L-glutamyl-tRNAGln + ATP + H2O <=> L-glutamate + L-glutaminyl-tRNAgln + ADP + phosphate + H+
Glycine	glycine biosynthesis I	2.1.2.1	serine hydroxymethyltransferase	L-serine + tetrahydrofolate <=> glycine + 5;10-methylenetetrahydrofolate + H2O
Glycine	glycine biosynthesis II	1.4.4.2	Glycine dehydrogenase (decarboxylating)	Glycine + H-protein-lipoyllysine <=> H-protein-S-aminomethyldihydrolipoyllysine + CO(2)
Glycine	glycine biosynthesis III	2.6.1.44	alanine--glyoxylate aminotransferase 1	glyoxylate + L-alanine <=> glycine + pyruvate
Glycine	glycine biosynthesis IV	4.1.2.5	L-threonine aldolase	L-threonine <=> acetaldehyde + glycine
Glycine	glycine biosynthesis IV	4.1.2.48	low-specificity L-threonine aldolase	L-threonine <=> acetaldehyde + glycine
Histidine	histidine biosynthesis	1.1.1.23	histidinal dehydrogenase / histidinol dehydrogenase	histidinol + 2 NAD+ + H2O = L-histidine + 2 NADH + 3 H+
Isoleucine	isoleucine biosynthesis I; I (from threonine); II; III; IV; V	2.6.1.42	branched-chain-amino-acid transaminase	L-isoleucine + 2-oxoglutarate <=> L-glutamate + (S)-3-methyl-2-oxopentanoate
Leucine	leucine biosynthesis	2.6.1.6	leucine transaminase	L-leucine + 2-oxoglutarate = 4-methyl-2-oxopentanoate + L-glutamate
Leucine	leucine biosynthesis	2.6.1.42	branched-chain-amino-acid transaminase	L-leucine + 2-oxoglutarate = 4-methyl-2-oxopentanoate + L-glutamate
Lysine	lysine biosynthesis I; II; III; VI	4.1.1.20	diaminopimelate decarboxylase	meso-diaminopimelate + H+ <=> CO2 + L-lysine
Lysine	lysine biosynthesis IV	1.5.1.7	saccharopine dehydrogenase (NAD+; L-lysine-forming)	L-saccharopine + NAD+ + H2O <=> 2-oxoglutarate + L-lysine + NADH + H+

Lysine	lysine biosynthesis V		N2-acetyl-L-lysine deacetylase	N2-acetyl-L-Lysine + H2O <=> L-lysine + acetate
Methionine	methionine biosynthesis I; II; III; methionine salvage II (mammalia)	2.1.1.13	methionine synthase	L-homocysteine + 5-methyl-tetrahydrofolate <=> L-methionine + tetrahydrofolate
Methionine	methionine biosynthesis I; III	2.1.1.14	methionine synthase	L-homocysteine + 5-methyltetrahydropteroyltri-L-glutamate + 4 H+ <=> L-methionine + tetrahydropteroyl tri-L-glutamate
Methionine	methionine biosynthesis II; S-methylmethionine cycle	2.1.1.10	homocysteine S-methyltransferase	S-methyl-L-methionine + L-homocysteine <=> 2 L-methionine + H+
Methionine	methionine salvage I (bacteria and plants); Yang cycle	2.6.1.88	methionine-oxo-acid transaminase	L-methionine + a 2-oxo carboxylate <=> 2-oxo-4-methylthiobutanoate + a standard _ amino acid
Methionine	methionine salvage II (mammalia)	2.1.1.5	betaine-homocysteine S-methyltransferase	L-homocysteine + glycine betaine <=> L-methionine + dimethylglycine
Methionine	S-methyl-5-thio-gamma-D-ribose 1-phosphate degradation II	2.5.1.49	O-acetylhomoserine aminocarboxypropyl transferase	O-acetyl-L-homoserine + methanethiol <=> acetate + L-methionine + H+
Phenylalanine	phenylalanine biosynthesis I	2.6.1.57	branched-chain amino-acid aminotransferase	2-keto-phenylpyruvate + L-glutamate <=> L-phenylalanine + 2-oxoglutarate
Phenylalanine	phenylalanine biosynthesis II	4.2.1.91	arogenate dehydratase	L-arogenate + H+ <=> CO2 + L-phenylalanine + H2O
Proline	proline biosynthesis I; II (from arginine); III; arginine degradation VI (arginase 2 pathway)	1.5.1.2	pyrroline-5-carboxylate reductase	(S)-1-pyrroline-5-carboxylate + NAD(P)H + 2 H+ <=> L-proline + NAD(P)+
Proline	proline biosynthesis IV	1.5.1.1	Pyrroline-2-carboxylate reductase	L-proline + NAD(P)(+) <=> 1-pyrroline-2-carboxylate + NAD(P)H
Serine	serine biosynthesis	3.1.3.3	phosphoserine phosphatase	3-phospho-L-serine + H2O <=> L-serine + phosphate
Threonine	threonine biosynthesis; threonine biosynthesis from homoserine	4.2.3.1	threonine synthase	O-phospho-L-homoserine + H2O <=> L-threonine + phosphate
Tryptophan	tryptophan biosynthesis	4.2.1.122	Tryptophan synthase (indole-salvaging)	L-serine + indole <=> L-tryptophan + H(2)O
Tryptophan	tryptophan biosynthesis	4.2.1.20	Tryptophan synthase	L-serine + 1-C-(indol-3-yl)glycerol 3-phosphate <=> L-tryptophan + D-glyceraldehyde 3-phosphate + H(2)O
Tyrosine	tyrosine biosynthesis I	2.6.1.5	tyrosine transaminase	L-tyrosine + 2-oxoglutarate <=> 4-hydroxyphenylpyruvate + L-glutamate

Tyrosine	tyrosine biosynthesis I	2.6.1.57	aromatic-amino-acid transaminase	L-tyrosine + 2-oxoglutarate <=> 4-hydroxyphenylpyruvate + L-glutamate
Tyrosine	tyrosine biosynthesis II	1.3.1.78	arogenate dehydrogenase	L-arogenate + NADP+ <=> L-tyrosine + CO2 + NADPH
Tyrosine	tyrosine biosynthesis III	1.3.1.43	arogenate dehydrogenase	L-arogenate + NAD+ <=> L-tyrosine + CO2 + NADH
Tyrosine	tyrosine biosynthesis IV	1.14.16.1	phenylalanine hydroxylase	tetrahydrobiopterin + L-phenylalanine + oxygen <=> L-tyrosine + 4_-hydroxy-tetrahydrobiopterin
Valine	valine biosynthesis	2.6.1.42	branched-chain-amino-acid transaminase	L-leucine + 2-oxoglutarate <=> 4-methyl-2-oxopentanoate + L-glutamate

The enzymes and enzyme information were curated by hand from the complete list of amino acid biosynthetic pathways found in the MetaCyc database.

[*] EC number was changed from 1.5.1.12 to 1.2.1.88, and may appear as either in UniProt
